# Study protocol: a multi-centre randomised study of induction chemotherapy followed by capecitabine ± nelfinavir with high- or standard-dose radiotherapy for locally advanced pancreatic cancer (SCALOP-2)

**DOI:** 10.1186/s12885-019-5307-z

**Published:** 2019-02-04

**Authors:** Victoria Y. Strauss, Rachel Shaw, Pradeep S. Virdee, Christopher N. Hurt, Elizabeth Ward, Bethan Tranter, Neel Patel, John Bridgewater, Philip Parsons, Ganesh Radhakrishna, Eric O’Neill, David Sebag-Montefiore, Maria Hawkins, Pippa G. Corrie, Timothy Maughan, Somnath Mukherjee

**Affiliations:** 1 Centre for Statistics in Medicine, University of Oxford, Oxford, UK; 2 Oncology Clinical Trials Office, University of Oxford, Oxford, UK; 3 Centre for Trials Research, Cardiff University, Cardiff, UK; 4 Clinical Trials and Evaluation Unit, Bristol Royal Infirmary, Bristol, UK; 5 Pharmacy Department, Velindre Cancer Centre, Velindre NHS University Trust, Cardiff, UK; 6 Department of Radiology, Oxford University Hospitals NHS Foundation Trust, Churchill Hospital, Oxford, UK; 7 Department of Oncology, University College London Hospitals, London, UK; 8 Cardiff NCRI RTTQA group, Department of Medical Physics, Velindre Cancer Centre, Cardiff, UK; 9 Oncology Department, The Christie NHS Foundation Trust, Wilmslow Road, Manchester, UK; 10 Department of Oncology, University of Oxford, CRUK MRC Oxford Institute for Radiation Oncology, Oxford, UK; 11 University of Leeds, Leeds Cancer Centre, St James’s University Hospital, Leeds, UK; 12 Cambridge Cancer Centre, Addenbrooke’s Hospital, Cambridge, UK

**Keywords:** Locally advanced pancreatic cancer (LAPC), Chemoradiation, Gemcitabine and nab-paclitaxel (GEMABX), Nelfinavir

## Abstract

**Background:**

Induction chemotherapy followed by chemoradiation is a treatment option for patients with locally advanced pancreatic cancer (LAPC). However, overall survival is comparable to chemotherapy alone and local progression occurs in nearly half of all patients, suggesting chemoradiation strategies should be optimised. SCALOP-2 is a randomised phase II trial testing the role of radiotherapy dose escalation and/or the addition of the radiosensitiser nelfinavir, following induction chemotherapy of gemcitabine and nab-paclitaxel (GEMABX). A safety run-in phase (stage 1) established the nelfinavir dose to administer with chemoradiation in the randomised phase (stage 2).

**Methods:**

Patients with locally advanced, inoperable, non-metastatic pancreatic adenocarcinoma receive three cycles of induction GEMABX chemotherapy prior to radiological assessment. Those with stable/responding disease are eligible for further trial treatment. In Stage 1, participants received one further cycle of GEMABX followed by capecitabine-chemoradiation with escalating doses of nelfinavir in a rolling-six design. Stage 2 aims to register 262 and randomise 170 patients with responding/stable disease to one of five arms: capecitabine with high- (arms C + D) or standard-dose (arms A + B) radiotherapy with (arms A + C) or without (arms B + D) nelfinavir, or three more cycles of GEMABX (arm E). Participants allocated to the chemoradiation arms receive another cycle of GEMABX before chemoradiation begins. Co-primary outcomes are 12-month overall survival (radiotherapy dose-escalation question) and progression-free survival (nelfinavir question). Secondary outcomes include toxicity, quality of life, disease response rate, resection rate, treatment compliance, and CA19–9 response. SCALOP-2 incorporates a detailed radiotherapy quality assurance programme.

**Discussion:**

SCALOP-2 aims to optimise chemoradiation in LAPC and incorporates a modern induction regimen.

**Trial registration:**

Eudract No: 2013–004968-56; ClinicalTrials.gov: NCT02024009.

## Background

Consolidation chemoradiation following induction chemotherapy is a treatment option for locally advanced pancreatic cancer (LAPC). The radiosensitiser capecitabine is commonly combined with radiotherapy doses of 50.4–54 Gy in 28–30 fractions [1]. However, current chemoradiation regimens have not demonstrated superior overall survival (OS) over chemotherapy alone [2, 3], local failure occurs in 40–60% of cases despite chemoradiation [4–6], and tumours are rarely down-staged to resectibility [1] or achieve pathological complete responses [5, 7]. Chemoradiation must be optimised to improve these outcomes, particularly as studies have shown that nearly one-third of patients with LAPC die due to local progression rather than systemic spread [8], and local failure may be a predictor of early mortality (hazard ratio [HR] = 2.15; 95% confidence interval (CI): 1.23–3.75, [4]).

### Rationale and feasibility of radiotherapy dose escalation

Pancreatic cancer is a radioresistant tumour surrounded by at-risk radiosensitive organs: the gastrointestinal tract, liver, and kidneys. A radiation dose range of 50.4–54 Gy in 28–30 fractions has traditionally been used to compromise between efficacy and toxicity. Although radiotherapy dose escalation remains challenging, modern-day technical radiotherapy advancements like intensity-modulated radiotherapy (IMRT) and image-guided radiotherapy allow precise delivery of radiotherapy while relatively sparing the organs at risk [9–11].

A small retrospective study in LAPC patients showed that a radiotherapy dose ≥54 Gy, versus < 54 Gy, was associated with improved OS (11.3 months vs 6.8 months, *p* = 0.089) [12]. A phase I/II radiotherapy dose-intensification study explored escalating the dose per fraction from 50 to 60 Gy in 25 fractions, concurrent with gemcitabine 1000 mg/m^2^ weekly in 50 patients with LAPC [9]. The study recommended a phase II dose of 55 Gy in 25 fractions and reported a median OS time of 14.8 months (95% CI: 12.6–22.2) and two-year OS rate of 30% (95% CI: 17–45%). Twelve participants underwent resection with a median OS time of 32 months. A planning study demonstrated that the radiation dose can be escalated to 72 Gy in 36 fractions using IMRT or proton therapy, based on the relationship between the gross tumour volume (GTV) and gastrointestinal tract [13].

### Rationale and evidence for nelfinavir as a radiosensitiser

Nelfinavir is a human immunodeficiency virus protease inhibitor traditionally used to manage acquired immune deficiency syndrome. Preclinical data has shown that nelfinavir and some other protease inhibitors inhibit phospho-inositol-3 kinase and downregulate Akt phosphorylation, leading to radiosensitisation [14–17]. As the Akt pathway is overactive in tumours but not normal tissue, radiosensitisation is selective without aggravating radiotherapy-mediated normal tissue damage. This radiosensitisation effect is even seen in KRAS-mutant pancreatic cell lines [14], which is highly relevant in clinical practice as > 90% of pancreatic tumours are KRAS-mutant. Inhibiting this pathway also normalises tumour vasculature, increases vascular flow and perfusion, and reduces hypoxia in tumour xenografts [18, 19].

Patients on nelfinavir who receive radiotherapy do not show increased acute toxicity [20]. Two early phase studies, ARC-I and ARC-II, prospectively tested nelfinavir with chemoradiation in pancreatic cancer. ARC-I was a phase I trial of 12 patients with LAPC who were treated with an upfront chemoradiation schedule of radiotherapy dose of 59.4 Gy in 33 fractions and 1250 mg nelfinavir twice-daily (from 3 days before the start of radiotherapy, until the final day of radiotherapy). Chemotherapy (30 mg/m^2^ cisplatin and 200–300 mg/m^2^ gemcitabine) was delivered on days 1, 8, 22, and 29 [21]. The study reported a Common Terminology Criteria for Adverse Events (CTCAE) grade 3–4 non-haematological toxicity rate of 16.7%. Half of the participants showed a metabolic complete response on FDG-PET, and a complete resection margin was achieved in six participants. The median OS time was 18 months. The recommended phase II dose for gemcitabine in the combination was 300 mg/m^2^.

ARC-II, a single-arm phase II study, tested the ARC-I regimen in another 23 patients [22] and reported promising outcomes (one-year OS rate 73.4, 90% CI: 54.5–85.5%; median OS time 17.4 months, 90% CI: 12.8–18.8). Although there was a high incidence of grade 3 or above gastrointestinal toxicity (nausea and vomiting 21.7%, diarrhoea 21.7%), nearly 75% of participants received the full radiotherapy dose and 87% received at least 80% of the intended nelfinavir dose. The gemcitabine+cisplatin combination chemotherapy and the large radiation field (which included elective nodal irradiation) may have contributed to gastrointestinal toxicity. Neither the gemcitabine+cisplatin combination regimen nor use of such large radiation fields are standard in current-day pancreatic chemoradiation.

Given the need for optimised chemoradiation in LAPC and nelfinavir’s promising radiosensitising effects in pre-clinical and early phase clinical trials, SCALOP-2 aims to optimise the chemoradiation regimen for managing inoperable LAPC by increasing the radiotherapy dose intensity and/or adding nelfinavir as an additional radiosensitiser in a randomised setting. As nelfinavir has not been combined with capecitabine-based chemoradiation for pancreatic cancer before, a nelfinavir dose-finding phase (stage 1) was included. Stage 2 investigates two co-primary questions: 1) does increasing the radiotherapy dose improve the 12-month OS rate (radiotherapy dose-escalation question), and 2) does adding nelfinavir to chemoradiation improve progression-free survival (PFS) (nelfinavir question). We report the study protocol (version 5.0, 31 July 2015 (Stage 1); version 6.0, 10 October 2017 (Stage 2)).

## Methods

### Study design and participants

**Fig. 1:**
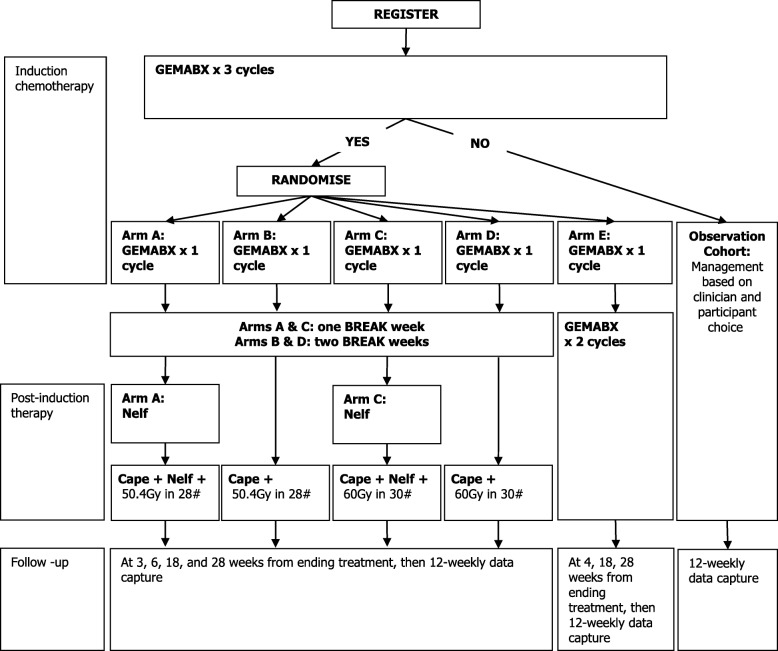
Schematic for Stage 2 of SCALOP-2. Legend: Stage 1 follows the flowchart as for Stage 2 Arm A. Nelfinavir dose is determined in Stage 1. Abbreviations: GEMABX = gemcitabine and nab-paclitaxel; Nelf = nelfinavir; Cape = capecitabine; Gy = Grays; # = fractions

Stage 1 of SCALOP-2 was a single-arm, dose-finding study of nelfinavir with capecitabine-chemoradiation using the rolling-six design [23]. Three nelfinavir dose levels were planned for investigation. Stage 2 commenced after the maximum tolerated dose of nelfinavir was determined in Stage 1. Stage 2 is a multi-centre, open-label, randomised 2 × 2 factorial + 1, phase II clinical trial investigating safety and efficacy (Fig. 1). Recruitment has opened and up to 30 UK centres are planned.

Entry criteria at registration include inoperable disease, histologically and/or cytologically proven LAPC, World Health Organisation (WHO) performance status of 0 or 1, and written informed consent (obtained at site via consent forms). After induction chemotherapy, participants are eligible for post-induction treatment if they have responding or stable disease after three cycles of chemotherapy by Response Evaluation Criteria in Solid Tumours (RECIST) 1.1, WHO performance status 0 or 1, adequate liver and renal function, ≤10% weight loss from baseline, and the tumour can be encompassed by a radically treatable radiotherapy volume (areas of known predilection for metastases). Table 1 lists the full entry criteria.

**Table 1:** Participant eligibility criteria for trial registration

Registration inclusion criteria	Registration exclusion criteria
1. Aged 18 years or over 2. Histologically or cytologically proven carcinoma of the pancreas 3. Locally advanced, non-metastatic inoperable disease as per National Comprehensive Cancer Network criteria. The following types of interventions are allowed: a. Palliative bypass procedure b. Common bile duct stenting 4. Primary pancreatic lesion 6 cm or less in diameter (taken from scan results) 5. World Health Organisation performance status 0–1 6. Adequate haematological function: neutrophils ≥1.5 × 10^9^/L, platelets ≥100 × 10^9^/L 7. Adequate liver function tests: a. Serum bilirubin ≤1.5 x ULN. In participants who have had a recent biliary drain and whose bilirubin is improving, a value of ≤3 x ULN is acceptable, however treatment should not start unless Bilirubin is ≤1.5 x ULN. b. AST and/or ALT ≤3 x ULN. 8. Adequate renal function: GFR ≥ 45 mL/min using a validated creatinine clearance calculation (e.g. Cockcroft-Gault, Wright formula, or as per local standard). If the calculated creatinine clearance is less than 45 mL/min, GFR should be assessed using a more formal method, e.g. 24 h clearance or an isotopic clearance method to confirm GFR ≥ 45 mL/min in order for the patient to be eligible for the study. This is optional and should only be carried if part of standard care. 9. Written informed consent obtained 10. Women of child-bearing potential must have negative serum or urine pregnancy test within 14 days prior to registration, must agree to use a highly effective contraception method during GEMABX treatment and for 30 days after last administration of GEMABX and to use an acceptable contraception method during chemoradiotherapy and for 6 months after completion of all treatment. 11. Male patients must be surgically sterile or must agree to use a condom during GEMABX treatment and for 90 days after last administration of GEMABX, and to use a condom during chemoradiotherapy and for three months after completion of chemoradiotherapy.	1. Primary resectable cancer of the pancreas. 2. Distant metastases 3. Pregnant or breast-feeding patients. 4. Any evidence of severe uncontrolled systemic diseases including uncontrolled coronary artery disease, myocardial infarction or stroke within the last 6 months, any major systemic or psychiatric co-morbidities or any other considerations that the investigator judges might impact on patient safety or protocol compliance and achievement of the study aims. 5. Previous malignancies in the preceding three years except for: a. In situ cancer of the uterine cervix b. Adequately treated basal cell skin carcinoma c. Adequately treated early stage non-pancreatic malignancy in complete remission for at least three years 6. Renal abnormalities including adult polycystic kidney disease or hydronephrosis or ipsilateral single kidney (i.e. functioning right kidney for head tumours; left kidney for tail tumours) that may preclude upper abdominal radiotherapy without damaging functional kidneys. 7. Previous radiotherapy to upper abdomen 8. Recurrent cancer following definitive pancreatic surgery 9. Lymphoma or neuroendocrine tumours of the pancreas 10. Known haemophilia A and B, chronic hepatitis type B or C. 11. Other experimental treatment six weeks or less prior to registration into this study (including chemotherapy and immunotherapy). 12. Known hypersensitivity to any of the investigational medicinal product or any of their excipients e.g. hypersensitivity to food products containing albumin. 13. Known dihydropyrimidine dehydrogenase deficiency 14. Known galactose intolerance, Lapp-lactose deficiency or glucose-galactose malabsorption 15. History of severe unexpected reaction to fluoropyrimidine therapies 16. If the following concomitant medications cannot be discontinued temporarily during the CRT phase then the patients cannot enter the trial: a. Sorivudine and analogues e.g. brivudine b. Methotrexate. c. Allopurinol and dipyridamole 17. Known HIV positive disease (but routine screening for HIV is not required)

*abbreviations: GEMABX* gemcitabine and nab-paclitaxel

### Randomisation and stratification

For stage 2, participants eligible for post-induction therapy are randomised in a 1:1:1:1:1 ratio to one of five treatment arms, using minimisation with a random element. Minimisation factors are centre, WHO performance status (0 or 1), and disease location (head or body/tail). Randomisation is performed centrally by the Oncology Clinical Trials Office (OCTO), University of Oxford, using a computer-based algorithm to conceal allocation and assigned via the OpenClinica database system.

### Interventions

#### Induction gemcitabine and nab-paclitaxel chemotherapy

All registered patients receive three cycles of gemcitabine and nab-paclitaxel (GEMABX) induction chemotherapy: 125 mg/m^2^ nab-paclitaxel intravenously for 30 min, then 1000 mg/m^2^ gemcitabine intravenously for 30 min, both on day 1, 8, and 15 of a 28-day cycle. Those eligible for post-induction therapy have a fourth cycle of GEMABX chemotherapy whilst radiotherapy is planned. Participants ineligible for post-induction therapy are treated at the investigator’s discretion and continue to contribute treatment and outcome data.

#### Post-induction therapy

Stage 1 participants received 50.4 Gy radiotherapy in 28 fractions over 5.5 weeks, with capecitabine (830 mg/m^2^ twice-daily taken orally on radiotherapy days) and nelfinavir. Nelfinavir was started 7 days before radiotherapy and taken orally twice-daily until the last day of chemoradiation. The nelfinavir dose depended on the assigned dose cohort: 750 mg, 1000 mg (the starting dose), or 1250 mg. The dose level was assigned by the safety review committee, following the rolling-six design. If radiotherapy was interrupted for reasons other than weekends, nelfinavir was also interrupted for that time. Nelfinavir and capecitabine compliance was monitored by review of patient diary cards.

Stage 2 participants receive post-induction therapy as per their allocated randomised arm. Participants in arms A and B receive 50.4 Gy radiotherapy in 28 fractions over 5.5 weeks, and in arms C and D 60 Gy radiotherapy in 30 fractions over 6 weeks. Participants in arms A-D also receive 830 mg/m^2^ capecitabine twice-daily taken orally on radiotherapy days. Participants in arms A and C also receive 1250 mg nelfinavir twice-daily (the dose determined in stage 1). Participants in arm E do not receive chemoradiation, but continue GEMABX chemotherapy (total 6 cycles). Nelfinavir and capecitabine compliance will be monitored by review of patient diary cards.

#### Radiotherapy

The GTV includes macroscopic pancreatic tumours with nodes > 1 cm on the short axis diameter. Prophylactic nodal irradiation is not acceptable. 4D planning is preferred, in which a composite GTV (GTV_C) is created from volumes outlined on the 3D CT scan and the 4D scan’s inhale and exhale phases. The clinical target volume (CTV_4D) is an expansion of 0.5 cm around the GTV_C, edited off the gastrointestinal tract. The planned target volume for the standard-dose arm (PTV5040) is a 0.5 cm expansion around the CTV_4D. For the high-dose arm, a PTV5400 (volume treated to 54 Gy, which is the CTV_4D with a 0.5 cm circumferential margin) and a simultaneous integrated boost (SIB) volume (PTV6000, identical to GTV_C) are created. If the 4D scan is not undertaken or fails, the CTV_3D is an expansion of 0.5 cm around the GTV_C, edited off the gastrointestinal tract. The PTV5040 and PTV5400 involve the CTV_3D with expansions of 0.5 cm cranial (exhale breath-hold*) or 1.5 cm (free breathing), 1.5 cm caudal, and 1.0 cm in ant-post and left-right direction. The SIB (PTV6000) will be GTV_3D + 0.5 cm expansion in all directions.

Participants receiving radiation at a standard dose receive 50.4 Gy in 28 fractions (1.8 Gy per fraction) to the PTV. They are treated once daily, five days per week, using photon beams of ≥6 MV. Stage 2 participants in the high-dose arms receive 54 Gy in 30 fractions (1.8 Gy per fraction) to the PTV (PTV5400) and the SIB will be delivered to the PTV6000 so that this volume receives a total dose of 60 Gy in 30 fractions (2 Gy per fraction). They are treated once daily, five days per week, using photon beams of ≥6 MV. IMRT is mandated for the high-dose arms and is preferred over 3D conformal radiotherapy for the standard-dose arms. Table 2 gives radiotherapy dose constraints.

**Table 2:** Radiotherapy dose constraints

Description	Naming Convention	Variable	Optimal	Mandatory
PTV High dose radiotherapy arms	PTV6000	D99%	≥ 95%	≥ 90% (≥ 83%)^a^
	PTV6000	D95%	≥ 97%	≥ 93% (≥ 90%)^a^
	PTV5400	D99%	≥ 95%	≥ 90%
	PTV5400	D95%	≥ 97%	≥ 93%
	PTV6000 & PTV5400	DMax (0.1 cc)	≤ 110%	≤ 115%
PTV Conventional dose radiotherapy arms	PTV5040	D99%	≥ 95%	≥ 90%
		D95%	≥ 97%	≥ 93%
		Dmax (0.1 cc)	≤ 105%.	≤ 107%
Kidney receiving higher dose	Kidney_R or Kidney_L	V20Gy	≤ 40%	≤ 45%
Combined Kidneys		V20Gy	≤ 30%	≤ 35%
Liver	Liver	V30Gy	–	≤ 30%
		Mean	≤ 28Gy	≤ 30Gy
Stomach	Stomach	Dmax (0.1 cc)	≤ 58Gy	≤ 60Gy
		V50Gy	< 5 cc	–
		V45Gy	< 75 cc	–
Small Bowel	SmallBowel	Dmax (0.1 cc)	≤ 58Gy	≤ 60Gy
		V50Gy	< 10 cc	–
		V15Gy	< 120 cc	–
Duodenum	Duodenum	Dmax (0.1 cc)	≤ 58Gy	≤ 60Gy
		V50Gy	< 10 cc	–
		V15Gy	< 60 cc	–
Spinal Cord PRV	SpinalCord_05	Dmax (0.1 cc)	–	≤ 45Gy

^a^When the gastrointestinal tract overlaps with a planned target volume, there is scope to reduce the dose in this region in order to prioritize gastrointestinal tract sparing. Abbreviations: *PTV* planned target volume

The radiotherapy trials quality assurance (RTTQA) programme was developed in collaboration with the National Cancer Research Institute RTTQA group. Pre-accrual, every participating centre must satisfactorily complete a benchmark exercise, including outlining and planning cases. The RTTQA team reviews these cases and provides detailed feedback. During the trial, the team prospectively reviews the first two outlines and the radiotherapy treatment plan for the first high and standard dose at each centre. The process is repeated for each centre if there is an issue with the outlines or radiotherapy plans, until there is a satisfactory submission. The volumes and plans for all subsequent cases will be retrospectively reviewed.

### Trial assessments

#### Screening

Participants are assessed at baseline for eligibility and all participants undergo a CT scan of the thorax, abdomen, and pelvis or a PET-CT scan (≤4 weeks but not > 6 weeks before starting induction chemotherapy). CA19–9 assessments are performed for all participants before treatment begins.

#### On-trial

Participants are assessed clinically prior to each cycle of chemotherapy and undergo a restaging CT scan after three cycles of chemotherapy to determine post-induction treatment eligibility. Post-induction participants receiving chemoradiation (stage 1 and arms A-D of stage 2) are assessed weekly during radiotherapy.

Assessments include weight, WHO performance status, and treatment-related toxicity, as per CTCAE V4.03. Haematology and biochemistry blood tests are undertaken ≤3 days before each chemotherapy dose and weekly during chemoradiation. CA19.9 assessments are made four times a week during chemotherapy. In the chemoradiation arms, CA19.9 assessments are done before starting and 4–6 weeks after finishing chemoradiation.

#### Post-treatment

Restaging scans are undertaken 4–6 weeks after completing treatment. Suitable patients are referred for surgery. Participants are followed-up every 10–12 weeks for the first year, with physical assessment, CA19.9, and CT scans at each visit. Participants are followed-up for at least 1 year, or until death (whichever is earlier). In each stage, follow-up will stop and the trial completed once the last patient on study reaches one year of follow-up.

#### Quality of life assessments

Stage 2 participants complete the European Organisation for Research and Treatment of Cancer self-reported questionnaires, QLQ-C30, PAN26, and EQ5D, at baseline; either the start of chemoradiation or cycle 5 of GEBAMX chemotherapy, as per randomised group; the post-treatment appointment; and follow-up appointments.

### Safety reporting

Any adverse event (AE) that occurs from consent (Stage 1) or the first trial treatment dose (Stage 2) up to 30 days after the last treatment dose are collected. AEs are graded using the CTCAE V4.03. In both Stages, any serious AE (SAE) from consent up to 30 days after the last treatment dose are collected. Serious adverse reactions (trial-treatment-related SAEs), will be collected until the end of follow-up. Suspected unexpected serious adverse reactions will be reported to the Medicines and Health Care Product Regulatory Agency (MHRA).

### Endpoints and outcome measures

#### Stage 1

Stage 1 determined the maximum tolerated dose of nelfinavir for use in stage 2. Three nelfinavir doses were tested: the starting dose of 1000 mg twice-daily was escalated to 1250 mg twice-daily or de-escalated to 750 mg twice-daily. The maximum tolerated dose was defined as the highest nelfinavir dose administered alongside chemoradiation at which none of three evaluable participants or no more than one of six evaluable participants experienced a dose-limiting toxicity (DLT).

A DLT was defined as per CTCAE V4.03 during chemoradiation and within 1 week post-chemoradiation as: any toxicity grade ≥ 4, any non-haematological grade 3 nelfinavir- or treatment-related AE or laboratory abnormality that the investigator deemed clinically significant, an inability to tolerate at least 20 fractions of radiotherapy due to an AE, or any SAE severe enough to halt radiotherapy for ≥14 days before recommencement (excluding events such as disease progression/stent blockage). DLTs were assessed at weekly clinic visits during and immediately after chemoradiation.

#### Stage 2

Stage 2 co-primary outcomes are 12-month OS rate (radiotherapy dose-escalation question) and PFS time (nelfinavir question). The 12-month OS rate is defined as the number of participants who die within 12 months of registration over the number of participants in a particular arm. PFS is defined as time (months) from registration to radiological/clinical progression or death, whichever occurs first. Participants who do not progress or die during the course of the study will be censored at their last known alive and progression-free date. Secondary outcomes include toxicity (as per CTCAE V4.03), quality of life, disease response rate over follow-up, resection rate over follow-up, treatment compliance, and CA19–9 response over follow-up.

### Sample size

Stage 1 used the rolling-six design with three dose cohorts and required up to 18 (6 per cohort) evaluable participants.

Stage 2 plans to randomise 170 participants (34 per arm). The sample size for the radiotherapy dose question (50.4 Gy [arms A + B, *n* = 68] vs 60 Gy [arms C + D, *n* = 68]) is based on the maximum of two binomial random variables and follows Dunnett’s ideas [24]. A 12-month OS rate of 60% (the bottom of the 95% CI for 12-month OS in SCALOP’s capecitabine-chemoradiation arm [1]) would not be large enough to warrant further investigation, whilst a rate of 80% is considered worthwhile. This design is based on a one-sided 5% significance level and a power of 90% of achieving significance if participants on one novel treatment have a survival rate of 80% and those on the second treatment a rate of 60%. A power greater than 90% will be achieved if both treatments have a worthwhile effect of 60%.

For the nelfinavir question, we assume median PFS time for participants not taking nelfinavir to be 12 months [1]. The sample size is calculated to provide 90% power to detect HR ≤ 0.65 with adding nelfinavir to chemoradiation (arms A + C, n = 68), compared with receiving chemoradiation without nelfinavir (arms B + D, *n* = 68), using a one-sided 20% significance level and assuming 5% loss to follow-up.

Sixty-five percent of SCALOP participants showed no evidence of radiological progression after three cycles of induction chemotherapy. We anticipated needing to recruit 27 participants to stage 1 to ensure there were up to 18 evaluable participants. We anticipate needing to recruit 262 participants to stage 2 to ensure 170 randomised (34 per arm).

### Statistical analysis

Stage 1 dose-level finding followed the rolling-six design. All participants with DLTs were evaluable for dose-escalation analysis. Participants without DLTs were evaluable if, during and ≤ 1 week after chemoradiation, they received at least 80% of the total intended (starting) dose of both nelfinavir and capecitabine, and at least 20 fractions of radiotherapy, and had completed the minimum safety evaluation requirements at each weekly clinic visit.

Stage 2 will use intention-to-treat based analyses, including all randomised participants. For the primary radiotherapy dose question: if 49 or more participants survive on one radiotherapy arm, that arm will be taken forward to a phase III trial. If both radiotherapy arms have 49 or more participants surviving, survival, proportion of participants completing the protocol dose radiotherapy, and toxicities observed will be taken into consideration to choose the treatment arm to take forward. If fewer than 49 of the 68 participants survive at 12 months on either treatment, no treatment will be taken forward to a phase III trial. For the primary nelfinavir question, a log-rank test will compare the survival curves between groups. The detailed statistical analysis plan gives full explanations of all planned analyses for primary, secondary, and tertiary aims.

### Regulatory and monitoring committees

SCALOP-2 is conducted in accordance with the regulatory requirements for clinical trials and standard operating procedures. Data management is via the online database system, OpenClinica, using electronic case report forms and anonymised data is stored confidentially at the Oxford Clinical Trials Research Unit. For stage 1, the safety review committee, consisting of independent clinical and medical oncologists, trial management representatives, and trial statisticians, monitored and reviewed the accumulated safety data and assigned doses. In stage 2, a data safety and monitoring committee, consisting of independent oncologists and a statistician, will be responsible for data and safety monitoring throughout the trial. It will first meet once 10 participants have completed high-dose radiotherapy treatment and 10 participants have completed nelfinavir treatment.

### Translational research

Blood samples and tumour biopsies are being collected for future translational work from participants who consent to take part in the translational part of the trial. Blood and biopsies are collected at baseline, the start of post-induction therapy, 4–6 weeks after ending post-induction therapy, and at disease progression, and stored at the Wales Cancer Bank, Cardiff University.

## Discussion

SCALOP-2 will investigate the benefit of GEMABX combination chemotherapy at induction, followed by different chemoradiation schedules evaluating standard- and high-dose radiotherapy with and without the addition of the radiosensitiser nelfinavir. This phase II 2 × 2 factorial + 1 design will allow the two chemoradiation research questions to be addressed efficiently. The inclusion of a chemotherapy-only arm, using internationally approved combination chemotherapy, will allow seamless progression to a phase III trial to formally compare the best-performing trial arm(s).

It is widely acknowledged that pancreatic cancer is a systemic disease. Chemoradiation’s role was brought into question after the LAP07 trial showed no survival benefit for chemoradiation over chemotherapy alone [3]. However, with greater understanding of tumour biology and improved imaging such as PET-CT, it may be possible to identify tumours that are truly localised and where loco-regional ablative therapies will still play an important role. Proposed molecular markers defining localised disease, like SMAD4, have been shown to be evaluable in both biopsy and cytology samples [25]. Understanding the mechanisms that lead to radio-resistance in pancreatic cancer and ways to overcome them will help to define radiation’s role in managing this disease. The ongoing sample collection from stage 2 participants will provide a large dataset with which to interrogate such translational questions. Trial results will be published in peer-reviewed journal(s) and presented at scientific conferences.

In SCALOP-2, management permission (“R&D approval”) was sought from all NHS organisations involved in the study, in accordance with NHS research governance arrangements, as listed here: Bristol University Hospitals NHS Foundation Trust; Cambridge University Hospitals NHS Foundation Trust; Colchester Hospital University NHS Foundation Trust; Hull and East Yorkshire Hospitals NHS Trust; Imperial College Healthcare NHS Trust; NHS Grampian; Norfolk and Norwich University Hospitals NHS Foundation Trust; North Middlesex University Hospital NHS Trust; Nottingham University Hospitals NHS Trust; Oxford University Hospitals NHS Foundation Trust; Royal Surrey County Hospital NHS Foundation Trust; Sheffield Teaching Hospitals NHS Foundation Trust; The Christie NHS Foundation Trust; The Leeds Teaching Hospitals NHS Trust; The Royal Free London NHS Foundation Trust; University College London Hospitals NHS Foundation Trust; University Hospitals Coventry and Warwickshire NHS Trust; University Hospitals Plymouth NHS Trust; Velindre NHS Trust. SCALOP-2 is open to international collaboration. Interested investigators are invited to contact the trials unit (octo-scalop-2@oncology.ox.ac.uk; 01865617078) for further discussion.

## Abbreviations

AE: Adverse event
CTCAE: Common Terminology Criteria for Adverse Events
DLT: Dose-limiting toxicity
GEMABX: Gemcitabine + nab-paclitaxel chemotherapy
GTV: Gross tumour volume
GTV_C: Composite gross tumour volume
HR: Hazard ratio
IMRT: Intensity-modulated radiotherapy
LAPC: Locally advanced pancreatic cancer
MHRA: Medicines and Health Care Product Regulatory Agency
OCTO: Oncology Clinical Trials Office
OS: Overall survival
PFS: Progression-free survival
RECIST: Response Evaluation Criteria in Solid Tumours
RTTQA: Radiotherapy trials quality assurance
SAE: Serious adverse event
SIB: Simultaneous integrated boost
WHO: World Health Organisation
